# A review of integrated heart failure care

**DOI:** 10.1017/S1463423618000312

**Published:** 2018-06-18

**Authors:** Julie MacInnes, Liz Williams

**Affiliations:** 1 Research Fellow, Integrated Care Research Unit, Centre for Health Services Studies, University of Kent, Canterbury, Kent, UK; 2 Senior Lecturer, School of Nursing, Canterbury Christ Church University, Canterbury, Kent, UK

**Keywords:** heart failure, integrated care, integrated review, primary care

## Abstract

**Aim:**

The aim of this integrative review is to determine the effectiveness of integrated heart failure (HF) care in terms of patient-, service- and resource-related outcomes, and to determine what model or characteristics of integrated care work best, for whom and in what contexts.

**Background:**

Integration of health and social care services is a significant driver in the development of better and more cost-effective health and social care systems in Europe and developed countries. As high users of health and social care services, considerable attention has been paid to the care of people with long-term conditions. HF is a progressive, prevalent and disabling condition, requiring complex management involving multiple health and social care agencies.

**Methods:**

An integrative review was conducted according to a framework by Whittemore and Knafl (2005). A literature search was undertaken using the databases: Medline, CINAHL, Embase, PsychINFO and the Cochrane Library, using key words of ‘heart failure’ OR ‘cardiac failure’ AND ‘integrated’ OR ‘multidisciplinary’ OR ‘interdisciplinary’ OR ‘multiprofessional’ OR ‘interprofessional’ OR ‘collaborative care’. Application of the inclusion and exclusion criteria resulted in 17 articles being included in the review. Articles were screened and coded for methodological quality according to a two-point criteria. Data were extracted using a template and analysed thematically.

**Findings:**

Integrated HF care results in enhanced quality of life (QoL), and improved symptom control and self-management. Reduced admission rates, reduced length of hospital stay, improved prescribing practices and better care co-ordination are also reported. There is more limited evidence for improved efficiency although overall costs may be reduced. Although findings are highly context dependent, key features of integrated HF models are: liaison between primary and secondary care services to facilitate planned discharge, early and medium term follow-up, multidisciplinary patient education and team working including shared professional education, and the development and implementation of comprehensive care pathways.

## Background literature

Significant policy initiatives in recent years have created a platform for integrated health, social care and support services in the United Kingdom and internationally. The Health and Social Care Act (HM Government, 2012) called for more integrated working between health and social care organisations in order to improve quality of care and patient outcomes and reduce inequalities. A mandate from the UK Government to the NHS promoted integration for the management of long-term conditions and Integrated Care and Support: Our Shared Commitment (Department of Health, 2013a) identified integrated care as a solution to the major pressures currently facing the health care system with a vision that integrated care will become the norm within the next five years. More recently, the Five Year Forward View (NHS England, 2014) called for greater integration of health and social care in order to deliver better care to patients. This includes hospitals working more closely with primary care, and more multidisciplinary teams operating in the community. The Care Act 2014 (HM Government, 2014) builds on existing government reforms to establish a new approach to adult social care. The Act promotes integration by introducing statutory requirements for local authorities to ensure the integration of social care and support with health provision. Moving forward, Goodwin (2017), describes integrated care as a fundamental design feature that will strengthen health care around the world.

Due to the growing interest in the integration of health and social care over the past decade, many different ways have emerged regarding how it operationalised and defined (The Nuffield Trust, 2011; National Voices, 2013). Integration may occur at macro, meso or micro levels. In the United Kingdom and other countries, ‘Accountable Care Organisations’ (ACOs) are formed at a macro level and describe a system of care that creates a single health and social care organisation which is contracted to deliver services to whole populations across large regions. At the meso level, new care models or so-called ‘Vanguard’ sites in the United Kingdom, describe groups of organisations in specific localities that collaborate to provide health and social care services to a defined population (The Kings Fund, 2018). Micro level integration is more about clinical and professional integration to enhance team performance (Billings and de Weger, 2015). For the purpose of this review, integrated care is considered at the meso level in which providers deliver integrated care for a particular group of people, and at the micro level in which providers deliver care for individual service users and their carers through care co-ordination, care planning and other approaches (Ham and Curry, 2011). The terms horizontal and vertical integration are also used in the literature. Horizontal integration refers to the alignment of health and social across one care setting, for example, primary care, whilst vertical integration occurs across primary, secondary, and community settings (Basi, 2014). However, it is acknowledged that these terms may not be used consistently between countries, where horizontal integration may be described as long-term care with the term ‘integrated care’ being reserved for services within health care systems.

Integration is a proposed solution for improving several chronic disease outcomes including those in cardiovascular disease (CVD). The Cardiovascular Disease Outcomes Strategy (Department of Health, 2013b) stresses the importance of integrating health and social care services to address the spectrum of conditions related to CVD. It states that, to achieve this, there must be further integration of care across the CVD pathways, including the development of new service models and a re-alignment of the interactions between hospital, primary and social care services (British Heart Foundation (BHF), 2015).

The term heart failure (HF) is one of a number of diseases that sit within the umbrella term of cardiovascular disease. HF is a common, progressive, life-limiting condition affecting around 550 000 people in the United Kingdom in 2014 (BHF, 2014). It is a disabling and distressing condition which can have a major effect on the quality of life of patients and their families. It is one of the commonest causes of all hospital admissions and the most common cause of admission in those aged over 65 years. The average length of hospital stay for a HF admission is 13 days and one in seven HF patients die in hospital or in the month following discharge. The typical cost per hospital admission episode has been estimated at £3796. HF accounts for 2% of the total NHS budget with 70% of these costs due to hospitalisation. It accounts for 1 million patient bed days per annum and 5% of all emergency admissions (BHF, 2014). In Europe, ~1–2% of the adult population have HF rising to ⩾10% among people >70 years of age. HF, therefore imposes a significant burden on individuals, society and the health and social care economies (ESC, 2016).

The clinical management of heart failure is based on established national and international guidelines (NICE, 2010; ESC, 2016). The BHF (2014) have called for an integrated approach to HF management with robust care pathways to meet patient needs from diagnosis through to end-of-life, including long-term follow-up, social support and palliative care.

## Methodology and methods

### Design

An integrative review methodology was used according to the approach of Whittemore and Knafl (2005). This consists of four stages: problem identification, literature search, data evaluation and data analysis. This methodology was chosen as it allows for the combination of diverse research designs using both qualitative and quantitative methods, to address a range of outcome measures.

### Problem identification

HF is defined as ‘*a complex clinical syndrome of symptoms and signs that suggest impairment of the heart as a pump supporting physiological circulation*’ (NICE, 2010: 19). The management of HF is a significant challenge for patients and their families and requires substantial financial resource, largely due to high rates of hospital admissions. Integrated care – both horizontal and vertical – has been identified as a model of service delivery with the potential to deliver quality care and improved patient outcomes. To date, there has been no review which considers the evidence on the effectiveness of integrated HF care in terms of outcomes. Given the diversity of integrated HF care models, a further aim is to address the question of what works, for whom and in what context?

### Literature search

A literature search was undertaken using the databases: Medline, CINAHL, Embase, PsychINFO and the Cochrane Library, using key words of ‘heart failure’ OR ‘cardiac failure’ AND ‘integrated’ OR ‘multidisciplinary’ OR ‘interdisciplinary’ OR ‘multiprofessional’ OR ‘interprofessional’ OR ‘collaborative care’. Limitations applied were English Language only and a date restriction of 2000–2017. The reference lists of included articles were hand searched for any further relevant papers. The *Journal of Integrated care* and the *International Journal of Integrated Care* were searched individually. A total of 161 articles were sourced which was reduced to 62 based on relevance to the topic.

### Inclusion and exclusion criteria

Articles were included if they related to adults with HF; described integrated or multidisciplinary practice involving a minimum of two organisations or professional groups; described a setting of primary care alone or primary care together with secondary care. Only studies which presented data on outcomes were included. Outcomes could be patient-, service-, or resource-related. All empirical study designs were included, using qualitative, quantitative and mixed methodologies.

Articles were excluded if they described CVD in which data relating to HF could not be isolated; if the practice of a single professional group was described; if the setting was exclusively secondary care or if outcomes were not reported. The two authors independently applied the inclusion and exclusion criteria to reach a final list of included articles.

**Figure 1:**
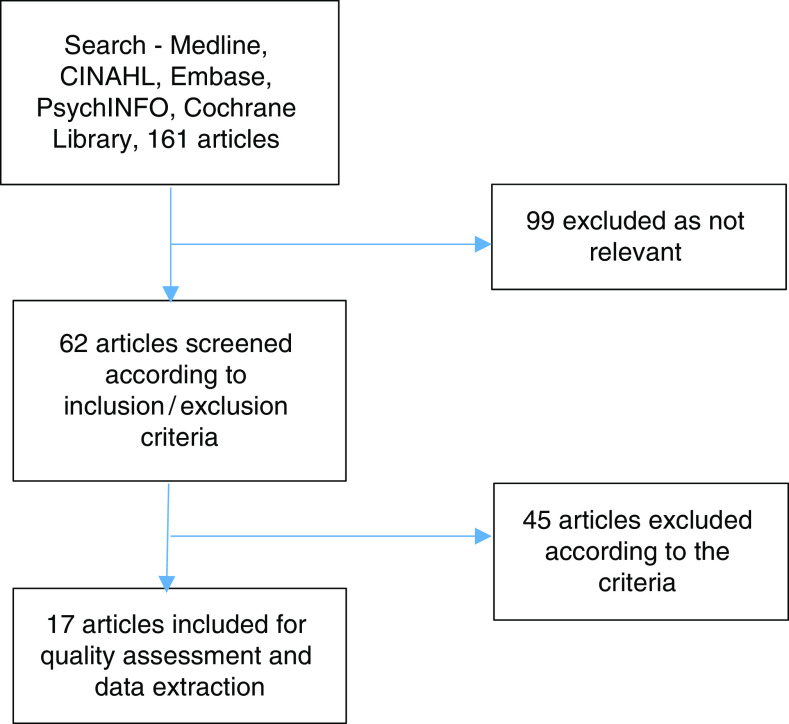
Selection of included articles

Application of the criteria resulted in 45 articles being excluded, primarily because they did not describe a model of integrated care or were review or editorial pieces. This resulted in a final list of 17 articles (Figure 1).

### Data evaluation

The included articles were screened for methodological quality. Given the diverse nature of primary sources, studies were coded according to a two-point criteria (high or low) relating to methodological rigour and relevance (Whittemore and Knafl, 2005). The authors independently carried out data evaluation. No articles were excluded on the basis of quality, rather this rating was used to evaluate the strength of the evidence at the point of data synthesis and discussion of findings.

### Data analysis

Data were extracted independently by the authors according to a template. A summary of the results is presented in Table 1. Outcomes were analysed thematically.

**Table 1:** Data extraction

Author	Aim(s)	Team	Integration/intervention	Number of patient	Study design	Outcomes
Asch *et al*. (2005), USA	To evaluate a collaborative model of care (Institute of Healthcare Improvements Breakthrough Series)	Physicians, nurses and other professionals	Three national, collaborative education sessions, based on the CCM. Teams implemented specific quality improvement interventions	*n*=489	Quasi-experimental	Significant improvement in the use of lipid-lowering medication and ACEI, education and counselling
British Heart Foundation (2015), UK	To improve identification, diagnosis, and management of HF	HFNS, GPs, cardiologists, social services	Five integrated care pilot sites. Various models including in-reach services to acute hospitals, discharge follow-up, home visits, telehealth, complex case management, rapid echo referral, implementation of a primary care bundle	Not stated	Case studies	Greater confidence and ability to self-manage; improvement in QoL and symptom control; patients better informed about their condition and prognosis Staff perceived reduced readmissions and length of hospital stays; more effective prescribing and up-titration; access to specialist telephone support and care; improved care co-ordination; improved identification and diagnosis of HF; more accurate disease registers; greater number of patients receiving reviews and having recorded NYHA status Increased job satisfaction, increased number of staff with specialist training, up-skilling of staff More cost-effective
Brannstrom and Boman (2014), Sweden	To evaluate an integrated palliative advanced home care and heart failure care (PREFER)	Specialist nurses, cardiologists, palliative care physicians, physiotherapists, occupational therapists	Collaboration between specialists in palliative and heart failure care	*n*=72	Prospective RCT	Intervention group had improved QoL (26% compared with 3% in the control group), total symptom burden improved by 18%, self-efficacy by 17%. NYHA improved by 39% compared with 10% in the control group 15 hospitalisations compared with 53 in the control group Increased nurse visits in the intervention group
Cawley and Grantham (2011), USA	To implement interventions to facilitate communication between clinicians in different care environments and to deliver a consistent approach to education	Interdisciplinary Joint HF Workgroup and ‘champions’ from different care settings and professional groups	Comprehensive strategies to link activities across the health system	Not stated	Case study	Enhanced communication, regular meetings, standardised education materials and tools for clinicians, promotion of cardiac rehabilitation, reduced readmission rates, increased completion of discharge forms, smoking cessation counselling, ACEI prescribing and access to telemonitoring, reduced duplication of services More patients stable or improved with medication, improved dyspnoea, enhanced confidence in self-management and goal-setting
Comin-Colet *et al*. (2014), Spain	To evaluate the feasibility and efficacy of an integrated HF management programme (IHFP)	Specialist nurses, cardiologists, other MDT members	Integrated HF management programme. Multidisciplinary approach based on the CCM	*n*=56 742	Comparative study	Increased quality of care, reduced mortality risk, lower risk of clinically related readmissions, lower risk of readmissions for HF in the IHFP
Cox *et al*. (2011), USA	To evaluate hospital to home (H2H) by preparing patients for self-management	Nurses, pharmacists, dietician, social care	Multidisciplinary patient education before discharge with follow-up case management by social care	*n*=56	Comparative study (pilot)	Readmission rates at 30 days reduced from 26.1% to 14.2% with H2H
Davidson *et al*. (2004), Australia	To evaluate a collaborative model of integrated palliative care and a HF disease management programme	Specialist HF and palliative care physicians and nurses, bereavement counsellor, GPs, occupational therapists, pastoral care workers, social workers, volunteers	Development of a systematic, multidisciplinary plan of care	*n*=121	Case study	48.8% of patients died at home; 8.3% required specific palliative care referral; decrease in hospital emergency presentations for HF
Del Sindaco *et al*. (2007), not stated	To determine the long-term efficacy of a HF disease management programme (DMP)	Cardiologist, nurses, GP	Discharge planning, education, therapy optimisation, early attention to signs and symptoms, intensive follow-up through hospital appointment, nurse phone call, GP visit	*n*=173	RCT	36% reduction in all-cause mortality; improvements in patient reported functional status and QoL Reduced all-cause and HF admissions; reduced length of stay; increased *β*-blocker prescription rates Reduced cost per patient with DMP
Doughty *et al*. (2002), New Zealand	To determine the effect of an integrated HF management programme	Nurses, GP, cardiologist	Clinic review early after discharge, education sessions, a personal diary, information booklets and clinic follow-up alternating between GP and HF clinic	*n*=197	Cluster RCT	Improved QoL; Fewer multiple admissions and associated reduction in bed days in the intervention group
Inglis *et al*. (2006), USA	To examine the long-term impact of a multidisciplinary home-based intervention compared with usual care	Specialist nurses, pharmacist, primary care physician, cardiologist	A structured home visit 7–14 days after discharge, referral to primary care physician or cardiologist if deterioration, medication management; long-term surveillance – telephone follow-up over six months	*n*=297	RCT	Median survival in intervention group almost twice that of control (40 versus 22 months); fewer deaths overall; prolonged event-free survival (7 versus 4 months) Reduced rates of readmission and length of hospital stays (14 versus 28 days) Increased cost-effectiveness
Johnson *et al*. (2012), UK	To assess the care received by patients with advanced HF in 2 integrated palliative/HF teams	HFNS, Marie Curie nurses, palliative care physicians and cardiologists	Cardiology-specialist palliative care MDTS, out-of-hours telephone advisory service, hospice-at-home	*n*=126	Prospective case studies	33% died in hospital with preferred place of death achieved for 61%; home death was more common with access to hospice-at-home and Marie Curie input Planning for end-of-life evident in 64% of cases with half referred to palliative care services
McDonald *et al*. (2002), Ireland	To determine whether multidisciplinary care of patients with HF reduces readmissions	Specialist nurses, dietician, cardiologist	In-patient education, plus outpatient education and telephone follow-up by the HFNS three days after discharge then weekly. Clinic follow-up at two and six weeks	*n*=93	RCT	Patients and carers had better understanding of HF and importance of diet and sodium restriction in the intervention group Fewer readmissions (3.9% versus 25.5%)
NHS Improvement (2011), UK	Improving HF services (final reports from the four national pilot sites)	Specialist HF and palliative care nurses and physicians	Integrated pathways to identify patients with HF in hospital, medication optimisation, discharge planning, liaison with and access to community palliative care services, advanced care planning, the use of an end-of-life trigger tool, joint training of HF and palliative care nurses	Not stated	Case studies	Increased proportion of patients discussing end-of-life (64% versus 21% before the intervention); more patients dying in their preferred place (55% versus 7%) Total readmission rates reduced by 42% and a reduction of 57% in <30 day readmissions; length of stay reduced from 12 to 4 days releasing 1249 bed days per year; increased use of palliative care services (3–31%); reduction in the number of patients dying in hospital (86% versus 47%), preferred place of death recorded (55% versus 12%)
Riegel *et al*. (2000), USA	To test the effect of a multidisciplinary disease management intervention in HF	Pharmacist, dietician, social worker, support group, specialist nurses, physicians	Education materials, in-hospital counselling, discharge assessment by a social worker, home visits by HFNS, telephone case management	*n*=240	Quasi-experimental	Days in hospital significantly lower in NYHA class II. Readmission rates lower by 17.6% in this class Acute care resources lower in class II, 62% total cost reduction
Sahlen *et al*. (2016), Sweden	To assess the cost effectiveness of person-centred integrated HF and palliative care (the PREFER trial)	Specialist nurses, cardiologist, palliative care physician, physiotherapist, occupational therapist	A structured MDT approach	*n*=72	Prospective RCT	Gain of 0.25 QUALYs; significant cost reduction due to a reduced need for hospital care even though staff costs are higher
Stewart and Horowitz (2002), USA	To assess the long-term effect of a multidisciplinary, home-based intervention for HF	Not described	Not described	*n*=297	RCT	Fewer deaths and prolonged event-free survival in the intervention group 78 fewer unplanned admissions and associated reduced costs
Stewart *et al*. (2012), Australia	A comparison of a home-based intervention (HBI) versus a clinic-based intervention – the WHICH trial	HFNS, nurses, cardiologist and primary care physician	Multidisciplinary HF management programme. Home visit by HFNS 7–14 days after discharge with detailed assessment and pharmacological and non-pharmacological management	*n*=280	Prospective RCT	Unplanned hospital admission or death occurred in 71% of HBI group versus 76% of CBI, at 12 months; 18% died in the HBI compared with 22% in the CBI; 67% of the HBI had 1 or more unplanned hospitalisation compared with 69% in the CBI; length of stay for unplanned admissions was significantly lower in the HBI Reduced costs of HBI due to fewer days in hospital

CCM=chronic care model; HFNS=heart failure nurse specialist; RCT=randomised controlled trial; MDT=multidisciplinary team; QUALY=quality adjusted life years.

Of the included articles, six were conducted in the United States, three in the United Kingdom, two in Sweden, two in Australia, one in New Zealand, one in Spain and one in the Republic of Ireland. One study did not state the country. The types of study were randomised controlled trials (*n*=8), case studies (*n*=5) and comparative designs (*n*=4). Two articles presented analysis from several different case studies (BHF, 2015; NHS Improvement, 2010). Narrative data from these case studies was presented individually and with an overarching evaluation. For the purpose of this review, the combined data were used so that the breadth of outcomes could be included. Most articles were assessed as high in terms of both methodological quality and relevance.

## Findings

A number of different types or models of integrated HF services were described, involving a range of professional groups.

### Vertical integration models

These included liaison between primary care and hospital staff through ‘out-reach’, for example, a follow-up telephone call by the hospital nurse following discharge (McDonald *et al*., 2002; Del Sindaco *et al*., 2007) or ‘in-reach’ where community nurses visited patients with HF before discharge (BHF, 2015). Vertical integration most commonly involved a limited number of professional groups – nurses and doctors. These were specialist staff such as cardiologists and heart failure nurse specialists or non-specialist staff such as hospital nurses and general practice physicians. Dieticians and pharmacists also contributed, usually by providing in-hospital education (Riegel *et al*., 2000; Cox *et al*., 2011). A wider multidisciplinary team, involving a ‘whole-systems’ approach to care is described by Cawley and Grantham (2011) and pilot studies within the NHS Improvement evaluation (2011). Here, comprehensive strategies link activities between primary and hospital care and represents the highest and most ambitious level of integration. Specific interventions associated with vertical integration models included pre-discharge education, discharge planning, early (within 14 days) community or clinic follow-up and medication optimisation.

### Horizontal integration models

Several studies focused on integrated HF and palliative care services at end-of-life. Integration was between HF and palliative care specialist nurses and physicians across different community settings such as home, hospices, nursing homes and community hospitals (Davidson *et al*., 2004; NHS Improvement, 2011; Johnson *et al*., 2012; Brannstrom and Boman, 2014; Sahlen *et al*., 2016). Horizontal integration models commonly consisted of multidisciplinary team working between doctors, nurses, pharmacists, dieticians, physiotherapists, occupational therapists, social services, bereavement counsellors, pastoral care workers and volunteers. Specific interventions associated with these models included multidisciplinary team meetings, joint professional education, telehealth, complex case management, rapid referral for diagnostic echocardiography, shared pathways of care and, for palliative care, out-of-hours advice and hospice-at-home services.

### Outcomes

#### Patient related

Improved quality of life (QoL) was widely reported (Doughty *et al*., 2002; Del Sindaco *et al*., 2007; Brannstrom and Boman, 2014; BHF, 2015) with better symptom control and improved functional status (Del Sindaco *et al*., 2007; Brannstrom and Boman, 2014; BHF, 2015). Self-management education resulted in improved patient knowledge and self-management ability (McDonald *et al*., 2002; Asch *et al*., 2005; Brannstrom and Boman, 2014; BHF, 2015). Studies also reported increased survival rates (Stewart and Horowitz, 2002; Inglis *et al*., 2006; Del Sindaco *et al*., 2007; Stewart *et al*., 2012; Comin-Colet *et al*., 2014) which was presented as a 36% reduction in all-cause mortality and median survival twice that of a control group.

#### Service related

Reduced hospital admissions/readmissions was the most commonly reported outcome (Riegel *et al*., 2000; Doughty *et al*., 2002; Stewart and Horowitz, 2002; Del Sindaco *et al*., 2007; Cawley and Grantham, 2011; Cox *et al*., 2011; NHS Improvement, 2011; Stewart *et al*., 2012; Brannstrom and Boman, 2014; Comin-Colet *et al*., 2014; BHF, 2015) along with a reduction in the length of hospital stay (Riegel *et al*., 2000; Inglis *et al*., 2006; Del Sindaco *et al*., 2007; NHS Improvement, 2011; Stewart *et al*., 2012; BHF, 2015). Readmission rates fell by between 11 and 57% with the most significant reductions in <30 day readmissions. Length of stay fell by between 8 and 14 days. A reduction in the number of hospital admissions and reduced length of stay was confined to patients with mild/moderate HF (NYHA, Class II) in one study, suggesting those with more severe HF may still require frequent admissions.

Improved prescribing practices were reported with more effective up-titration and prescription of β-blockers and ACE-inhibitors (Asch *et al*., 2005; Inglis *et al*. 2006; Del Sindaco *et al*., 2007; Cawley and Grantham, 2011; BHF, 2015). Better care co-ordination, comprehensive documentation and reduced duplication is cited by the BHF (2015) and Cawley and Grantham (2011). Earlier patient identification and diagnosis through, for example, rapid access to echocardiography was also reported (BHF, 2015). At end-of-life, a greater number of patients died at home or in their preferred place (Davidson *et al*. 2004; NHS Improvement, 2011; Johnson *et al*., 2012). This is an important quality indicator aligned to the End of Life Care Strategy (DH, 2009). Finally, greater satisfaction and up-skilling was reported by staff in one study (BHF, 2015).

#### Resource related

Studies by Riegel *et al*. (2000), Stewart and Horowitz (2002), Del Sindaco *et al*. (2007), Stewart *et al*. (2012) and Sahlen *et al*. (2016) all reported reduced costs associated with integrated HF care, although rarely is an economic analysis presented. Although staff costs may be increased, this is offset by reduced hospital admission rates and length of stay, and reduced indirect costs due to improved patient-related outcomes.

## Discussion

Frequently, multiple interventions are described as part of an integrated HF service which means it is difficult to determine which interventions have the greatest impact on what outcomes, in specific contexts. However, there are commonalities between the reviewed models which suggest that integrated HF systems which include some or all of these features may result in improved outcomes. These features are: liaison between primary and secondary care services to facilitate a planned discharge, early (<14 days) and medium term (6 months) follow-up, patient self-management education provided by a multidisciplinary team, medication optimisation, multidisciplinary team working; shared education and the development and implementation of comprehensive patient pathways across settings.

Jaarsma *et al*. (2013) developed a guide for home health in HF patients from a literature review, a survey of HF management programmes and expert opinion. They concluded that care should consist of integrated multidisciplinary working, patient and partner participation, the development of care plans with clear goals, patient education, self-care management, appropriate access to care and optimised treatment. The present literature review is consistent with this guide, although patient and partner participation has not been widely adopted.

Multidisciplinary teams most commonly consisted of doctors and nurses, both specialist and non-specialist. Dietician and pharmacist input is also cited, most specifically in providing patient education in relation to diet and medication management. This is not an unsurprising finding given the importance of a low sodium diet and fluid management and adherence to complex medication regimes (NICE, 2010; ESC, 2016). However, in general, there is an absence of other professional groups, most notably mental health professionals and social care staff. Integrated care in HF as in other services often remains health-dominated (Goodwin, 2017). This needs to be addressed if the ambition for integrated care is to be realised.

A few studies detailed either the severity or type of HF. Although Riegel *et al*. (2000) differentiated between New York Heart Association (NYHA) (1994) functional classifications (I-IV), in determining outcomes, the stage of the disease was not discussed in other studies beyond stating that HF was chronic or advanced (terminal). Similarly, the type or aetiology of HF was infrequently stated. Given that the management and prognosis for left ventricular systolic dysfunction and right-sided heart failure, for example, are significantly different (NICE, 2010; ESC, 2016), it seems likely that integrated care models will produce different outcomes in these specific populations. It therefore, remains unclear whether the positive outcomes cited are confined to different levels of severity or types of HF.

The search for effectiveness and clearly defined patient outcomes through integrated care service delivery in general remains elusive, due to patient multi-pathology, multiple integrated care configurations and methodological design challenges (Billings and Leichsenring, 2014). However this review has demonstrated that focusing on a single disease can cast a sharper spotlight on pathway solutions. There are relatively well-developed pathways for palliative and end-of-life care for cancer patients but these are less well developed in other diseases such as HF and chronic obstructive pulmonary disease. However, this review has indicated that integrated HF and palliative care at end-of-life can produce significantly improved patient outcomes.

## Conclusion

The management of HF presents complex challenges for individuals, their families and caregivers, society and health and social care economies. To address this, a number of countries have implemented integrated HF services either involving multidisciplinary team working in primary and community care, or across primary, community and secondary care settings. Multidisciplinary teams most frequently include specialist nurses and doctors but also pharmacists and dieticians. There is good evidence to suggest integrated HF care produces better outcomes for patients and improved care co-ordination across services and organisations. There may also be a reduction in costs, primarily due to reduced hospital admission rates and length of stay. A number of features of integrated HF care models are identified which are most likely to result in improved outcomes. These include liaison between primary and secondary care to facilitate planned discharge, early and medium term follow-up, multidisciplinary patient education and team working including shared professional education, medication optimisation and the development and implementation of comprehensive care pathways across settings.

## Limitations of the review

There is considerable heterogeneity of integration models, methodologies and outcomes so that meta-analysis is not possible. However, an integrative review does allow conclusions to be drawn. Only articles published in English were included which may limit both the scope and the generalisability of findings. Although some authors reported the challenges of implementing integrated HF care, outcomes were exclusively positive which may suggest some publication bias.

## Implications for policy and practice

Service commissioners and provider organisations should develop integrated health and social care services for HF, including at end-of-life. This includes the development and implementation of agreed care pathways spanning primary and secondary care with consideration given to a core set of interventions. The effectiveness of these pathways, within specific contexts, should be evaluated. There is not a one-size fits all model; effective integration depends on the availability of resources and the context within which health and social care systems operate. Patients and carers should be involved in the co-design of services.
